# Effectiveness of nurse-led dietary modification recommendations in the management of defecation dysfunction among patients with rectal cancer after sphincter-saving surgery: a scoping review

**DOI:** 10.3389/fsurg.2026.1761105

**Published:** 2026-06-11

**Authors:** Liu Wen, Qi Bi Rong, Cao Qiu jun, Wen Tao Tang, Xu Jian Min, Zhu Ai yong

**Affiliations:** 1 School of Nursing, Shanghai University of Medicine and Health Sciences, Shanghai, China; 2 Department of General Surgery, Zhongshan Hospital, Fudan University, Shanghai, China

**Keywords:** dietary modification, nurse-led intervention, rectal cancer, scoping review, sphincter-saving surgery

## Abstract

**Aim:**

Different degrees of defecation dysfunction emerge after sphincter-saving surgery. It is essential to implement dietary management interventions to manage defecation dysfunction. This scoping review aimed to map the existing literature on nurse-led dietary modification recommendations for defecation dysfunction management and highlight any gaps in the literature.

**Design:**

A systematic scoping review model was applied.

**Methods:**

Four comprehensive databases were searched (01 November 2014 to 26 May 2025) and records were independently screened by two researchers using eligibility criteria. Articles were included if they were published in a peer-reviewed journal, employed quantitative research designs, and described a nurse-led dietary modification intervention for patients after sphincter-saving surgery. Data from the included studies were charted using a purpose-designed template, and data charting included participant characteristics, study methodology, information about the nurse-led dietary modification intervention, and the effectiveness of the intervention.

**Results:**

Five publications met the study inclusion criteria: four randomized controlled pilot studies and one population-based, before-and-after pilot study. The studies varied in terms of intervention components, timing of delivery, and delivery mode. Two studies specifically focused on the effects of dietary modification programs, while the other three studies listed dietary management as one of the comprehensive nurse-led interventions. Nurse-led dietary recommendations included dietary fiber management, a diet diary, symptom-specific dietary modifications, and fiber supplement usage.

**Patient or public contribution:**

Nurse-led dietary modification recommendations had different degrees of effectiveness in improving defecation dysfunction in this review. Further research regarding remote and precise nurse-led dietary management is warranted, and a more standardized, evidence-based nurse-led dietary modification program could be provided for clinical nursing care.

**Systematic Review Registration:**

PROSPERO ChiCTR2400082167.

## Highlights

### What is already known

- Dietary intake factors are closely related to defecation dysfunction in patients after sphincter-saving surgery and dietary modification plays an important role in the self-management of defecation dysfunction.
- The increasing demand for scientific nursing care in rectal cancer calls for evidence-based nursing interventions.
- No review of nurse-led dietary modification interventions for patients with rectal cancer and defecation dysfunction is currently available.

### What this paper adds

- This scoping review contributes to a better understanding of the evidence base on dietary management in patients who undergo sphincter-saving surgery.
- This review indicates that nurse-led dietary modifications have different degrees of effectiveness in improving defecation dysfunction after sphincter-saving surgery.
- It is highly recommended that future high-quality intervention studies be conducted to develop a standardized, evidence-based nurse-led dietary modification program for the management of defecation dysfunction and to promote remote, precise, and scientific clinical nursing care.

## 1 Introduction

Colorectal cancer is one of the most common gastrointestinal malignancies in the world (1). According to the 2022 edition of the Global Cancer Statistics Report, the incidence and mortality of colorectal cancer occupy third and second place, respectively, among all malignant tumors worldwide (2). In China, with rapid social and economic development, residents’ lifestyles and diet structures are constantly changing, and the incidence of colorectal cancer has shown a significant upward trend in recent years (3). In 2020, the incidence of colorectal cancer in China rose to second place due to the large population base in China (1). Among the cases of colorectal cancer in China, the incidence of rectal cancer is higher than that of colon cancer, and patients with low rectal cancer account for approximately 70%–80% of the total number of patients with rectal cancer (4, 5).

At present, radical surgical resection is still the most important method for the treatment of rectal cancer (6). In recent years, with the continuous deepening of research on rectal cancer and the constant updating of surgical instruments and treatment methods, sphincter-saving surgery has gradually become the preferred surgical method for rectal tumor resection, accounting for more than 70% of the total number of rectal cancer operations (7, 8). This allows the preservation of the anus of many patients with rectal cancer who undergo radical surgery. However, retaining the anus does not mean that the normal bowel function of the anus can be completely preserved after surgery. Many patients with rectal cancer have varying degrees of defecation dysfunction symptoms after sphincter-saving surgery, including increased frequency of defecation, rapid stool, frequent stool, inadequate stool, liquid stool, gas stool, fecal incontinence, and other symptoms, which are collectively also called low anterior resection syndrome (LARS) (9, 10). According to research reports, the incidence of defecation dysfunction after sphincter-saving surgery is 90%, and approximately 20%–50% of patients have severe defecation dysfunction (9–12). Some patients’ defecation dysfunction continues until 15 years after surgery (13, 14). Prolonged defecation dysfunction is a significant burden on patients and seriously affects their postoperative quality of life (14–16). Therefore, alleviating the symptoms of postoperative defecation dysfunction in patients with rectal cancer has become a problem that needs urgent attention and a solution.

At present, the control of defecation dysfunction in patients who undergo sphincter-saving surgery mainly relies on patient self-management, including diet adjustment, use of adjuvant drugs, adjustment of social activities, and psychological adjustment (17–19). There is a close relationship between dietary intake and defecation function, and different food components can directly affect intestinal motility, regulate intestinal flora, and affect stool morphology and composition (20). Some studies have reported that patients believe that the intake of certain foods will affect the occurrence of defecation dysfunction, and some patients will adjust their diet after surgery to control defecation dysfunction symptoms, including changing their eating time and the type of food eaten (17, 21, 22).

Our research group found in a previous study that there was a close correlation between dietary intake and defecation dysfunction, and the dietary intake of patients with rectal cancer after sphincter-saving surgery changes significantly compared with that before surgery; however, this change was not entirely conducive to the relief of defecation dysfunction. The daily intake of livestock and poultry meat, fats, milk and dairy products, and vegetable foods within 6 months after surgery were the main dietary intake factors affecting the occurrence and relief of postoperative defecation dysfunction (23). Thus, it has been shown that reasonable and scientific patient dietary intake after surgery is of great significance for effectively controlling defecation dysfunction symptoms. However, current research on the relationship between dietary intake and defecation dysfunction is lacking.

Many patients recognize the importance of dietary management for defecation dysfunction, but in some previous studies, the patients generally reported a lack of dietary guidance after surgery. “Trial and error” was the most frequently mentioned method of diet self-management, which made it difficult to guarantee the scientific nature of the patients’ self-dietary management measures; thus, there are many difficulties and obstacles in the diet self-management process. Some patients pay too much attention to dietary changes and apply too many unnecessary dietary restrictions (18). In another study, although patients tried to change their diet after surgery, only one-third of the patients adopted a dietary pattern that was effective for controlling bowel dysfunction (24). Other studies have also found that problems such as malnutrition, low immunity, and an unbalanced diet emerge during patients’ diet self-management due to the lack of scientific rigor.

All these problems show that scientific diet management programs are very important. To the best of our knowledge, no professional nursing organizations have developed guidelines outlining recommendations for diet self-management for defecation dysfunction after sphincter-saving surgery. Research on a nurse-led intervention to effectively control defecation dysfunction is currently in the initial stages.

From a nursing perspective, patients discharged after sphincter-saving surgery require information and guidance regarding dietary management for defecation dysfunction. The dietary management recommendations described in existing studies are appropriate and within the scope of practice for nurses to use to guide patients. This scoping review aimed to map the existing literature on nurse-led dietary modification interventions to manage defecation dysfunction in patients after sphincter-saving surgery, and to identify any gaps in the literature.

The research questions for this scoping review were as follows: (1) What are the available nurse-led dietary modification recommendations for patients with rectal cancer after sphincter-saving surgery to control defecation dysfunction? (2) What are the main components, timing, and delivery modes of nurse-led dietary modification interventions? (3) What are the main findings relating to the interventions from the perspective of defecation dysfunction management among patients after sphincter-saving surgery?

## 2 Materials and methods

### 2.1 Design

Nursing recommendations for dietary modification strategies for defecation dysfunction are not uniformly described in the literature. A systematic scoping review model was applied. It is a useful way of examining the extent, range, and nature of a research area and of mapping current knowledge in areas that have limited randomized controlled trial evidence available (25). As no review of nurse-led dietary management recommendations has previously been conducted in patients with rectal cancer after sphincter-saving surgery, we adopted the JBI scoping review methodology (26) and used the Preferred Reporting Items for Systematic Reviews and Meta-Analyses extension for Scoping Reviews (PRISMA-ScR) guidelines (27). This study has been registered in the Chinese Clinical Trial Registry (https://www.chictr.org.cn/; the registration number is ChiCTR2400082167).

### 2.2 Inclusion and exclusion criteria

This systematic scoping review included articles describing patients with defecation dysfunction following sphincter-preserving anterior resection surgery. This review is limited to publications that investigated specific dietary modification recommendations within the scope of standard nursing practice. Inspired by Wright and Bell’s proposed definition of nursing interventions, we included interventions only when a nurse was accountable for delivering or providing the intervention and when the intervention occurred in the context of a nurse–patient relationship (28). English-language research studies that described symptom-specific, detailed nurse-led dietary modification recommendations after sphincter-saving surgery were included. To capture current nurse-led dietary modification interventions, studies published from 2014 onward were included. No restrictions on the nurses’ educational levels were applied.

Excluded from this review were protocols for research studies, systematic or scoping reviews, qualitative studies, and articles without full text available. Studies with interventions such as biofeedback, medicine use, and surgery were not excluded.

### 2.2 Search strategy

A search across four databases (Web of Science, Ovid Medline, PubMed, and EMBASE) was conducted in November 2024 and updated in May 2025. Weekly alerts were set up in the selected databases to inform the authors of any new publications that met the search criteria during the review process. Search terms were joined with Boolean operators (AND and OR) to include (“Low Anterior Resection Syndrome (Topic) OR bowel symptoms (Topic) OR bowel dysfunction (Topic) OR defecation dysfunction (Topic)”) AND (“rectal cancer (Topic) OR colorectal cancer (Topic)”) AND (“diet (Topic) OR intake (Topic) OR dietary (Topic) OR food (Topic)”). Medical Subject Headings and thesaurus options were utilized when appropriate. As the related literature in this area is limited, the terms “nursing” and “nurse-led” were not included in the search process, so that any potentially related studies were not excluded.

### 2.3 Study selection

**Figure 1:**
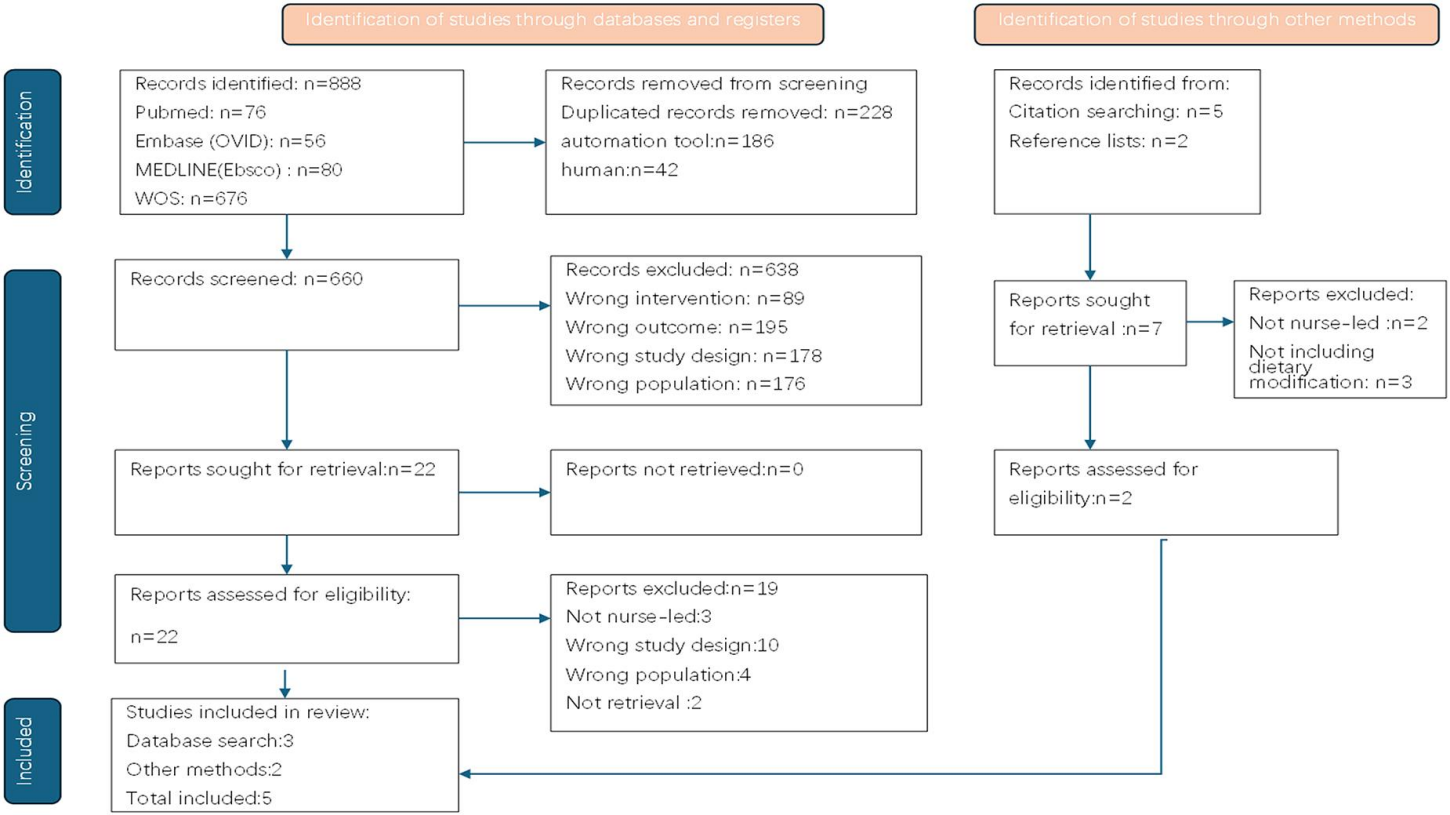
PRISMA 2020 flow diagram for new systematic reviews, including searches of databases, registers, and other sources.

The identified studies were collated and uploaded into EndNote (Version X9) and duplicates were removed. For screening, the results were imported into the RAYYAN system to sort the sources. The titles and abstracts were then screened for compliance with the inclusion criteria by two independent reviewers (WL and BQ). Next, the two reviewers assessed the full text of the selected studies in detail to identify compliance with the inclusion criteria. If any disagreements existed between the two reviewers during the study selection process, they would meet to discuss the inclusion of the studies until they reached an agreement. A PRISMA flow chart of the selection process is presented in Figure 1.

### 2.4 Data extraction process

Data were descriptively analyzed and presented narratively in relation to the guiding research questions. A purposefully designed template was used to record information from included studies about the author, timing, intervention components, design, sample, outcome measures, and key findings. The template was developed by the first author, circulated within the research team, and refined in an iterative process (29).

### 2.5 Ethical approval

This study has undergone full ethical review by the REDACTED Hospital Medical Ethics Committee and was approved on 23 January 2024 (B2024-019R).

## 3 Result

The initial database search was conducted on 1 November 2024, and a total of 888 records were retrieved. There were 660 records after removing duplicates using an automation tool (*n* = 228) and human identification (*n* = 42). After title and abstract screening, 638 articles were excluded as they did not meet the inclusion criteria (incorrect intervention, outcome, study design, and/or population). Moreover, seven records were identified from searching the citations and references of the articles. The full texts of 29 articles were read and discussed by the reviewers, which yielded 5 articles that met the inclusion criteria for this review (Figure 1). The results presented below are based on the five identified nursing interventions and a summary of the included studies is presented in Table 1.

**Table 1:** Summary of the included studies.

Author(s), year, country	Study design	Number of patients	Population	Aim of the study	Measurements of outcome and data collection
Tsui et al. (30), 2023, China, Taiwan	RCT, two parallel arms	Total: 66; EL: 34; routine: 32	Age 20–85; stages I–III; colorectal cancer	Effect of dietary fiber EL on post-op bowel symptoms	LARSS, Dietary Fiber Food Scale, SQTFFQ, BSRS-5; data collected at baseline and 1, 3, and 6 months
Sun et al. (31), 2024, US	RCT pilot (1:1)	Total: 93; AIMS-RC: 47; control: 46	Mean age 55.2; rectosigmoid/rectal cancer; treatment 6–24 months; minor-major LARS	Assess AIMS-RC for bowel dysfunction	Primary: BFI; secondary: LARS, QOL, HEI-2015, PROMIS, motivation, I-PANAS-SF
Dalsgaard et al. (32), 2021, Denmark	Population-based pilot	Total: 86	Mean age 65; treatment 10–20 months; major LARS; restorative resection	Nurse-led intervention for bowel dysfunction	LARSS score at baseline, first visit, and follow-up
Li et al. (33), 2022, China	Pilot RCT	Total: 95; intervention: 47; control: 48	Mean age 60.9; mid/low rectal cancer after SPS	Self-management program for bowel symptoms after SPS	LARSS, EORTC-QLQ-C30, BSSBQ; data collected at baseline and 3 and 6 months
Kim et al. (34), 2023, South Korea	Prospective, unblinded RCT	Total: 42; experimental: 21; control: 21	Mean age 57.6; male; rectal cancer; LAR or ileostomy repair	Bowel function improvement program for male patients	Primary: LARSS score (3 months); secondary: self-efficacy, EORTC-QLQ-CR29, and resource use; data collected at 1 and 3 months

Interventions		Control group		Key findings	
**Two stages:** **1. First stage (during hospital stay, before discharge)**: A one-time, 50-mine nurse-led session covering food categories, dietary fiber, bowel function, and an interactive food selection game using food cards. **2. Second stage (1 month after surgery**): A 30-min face-to-face nursing consultation focused on applying dietary knowledge to daily life, aiming to improve self-reflection and problem-solving skills.		Participants received a pamphlet to teach them about food appropriation after colorectal surgery		**Positive correlation:** EL during a hospital stay for colorectal surgery was linked to better postoperative bowel symptoms. **Associated benefits:** This improvement in bowel symptoms was further associated with higher dietary fiber intake, and reduced surgical site issues and emotional distress. **Key outcome:** Compared to the routine group, the EL group showed significantly greater improvement in bowel symptoms at both 1 and 3 months after surgery.	
Summary of the MAPS-guided, social cognitive theory intervention **Theoretical basis:** Social cognitive theory, guided by the MAPS model of behavior change. **Format:** 10 centrally administered telephone sessions over 17 weeks (average 27 min/session). **Materials:** Participants received an AIMS-RC resource manual before starting. **Session breakdown:** **Session 1** (within 10 days of randomization): Program introduction and manual overview. **Session 2** (1 week later): Coached to keep a food-symptom diary and set SMART goals. **Sessions 3–6** (weekly): Reviewed food-symptom diary and SMART goals. **Sessions 7–8** (every other week): Focused on reintroducing patient-identified foods using a 3-day schedule. **Sessions 9–10** (monthly): Reviewed progress and reinforced skills to build long-term self-efficacy.		Summary of the control group intervention (healthy living education) **Format:** 10 centrally administered telephone sessions over 17 weeks. **Average length:** 12.5 min (range 4–37 min). **Material:** Education resource manual used during sessions. **Session breakdown**: **Session 1** (within 10 days of randomization): Covered 10 health promotion topics based on national cancer survivorship guidelines (e.g., exercise, sun safety, sleep, food safety, skin care, bone health, clinical trials, online resources, and screening/surveillance). **Sessions 2–6:** Weekly calls. **Sessions 7–8:** Every other week calls. **Sessions 9–10:** Monthly calls.		**Primary outcome:** No statistically significant difference between groups in total bowel function score. **Exploratory endpoints:** The AIMS-RC group showed statistically significant improvements in LARS (*p* = 0.01) and the frequency subscale of the bowel function index (*p* = 0.03). **Acceptability:** The AIMS-RC group reported significantly higher study acceptability.	
**Approach:** Nurse-led personalized conservative treatment (PCT) in the clinic. **First visit:** Collected bowel dysfunction history; explained anatomy and effects of surgery/radiotherapy; provided dietary and toilet routine advice. Selectively added fiber supplements, rectal emptying aids, anti-diarrheals, laxatives, or pelvic floor training based on symptoms. **Follow-up (∼2 months):** Nurse-evaluated and optimized treatment by phone. Added basic treatments as needed and scheduled additional clinic visits or phone calls. In the case of an insufficient response, offered TAI and/or biofeedback. **Discharge:** When the patient is satisfied with the treatment. **Utilization and outcomes:** Median of one clinic visit (IQR 1–2) and two phone consultations (IQR 1–3); average of 3.7 treatment modalities (range 1–7); discharged after a median of 6 months (IQR 3–12).		NA		**LARS score:** Median decreased significantly from 37 to 31 (*P* < 0.001). **Major LARS prevalence:** Fell from 95% to 53% (*P* < 0.001).	
**Intervention (in addition to standard care):** A researcher delivered a 30–60 min face-to-face self-management program 2–3 weeks after surgery. **Follow-up:** 10–20 min calls or WeChat messages. Frequency: weekly for the first 4 weeks, then every 2 weeks until 6 months post-surgery. **Six self-management strategies for bowel dysfunction:** Therapeutic management, perianal skin care, dietary management, medical guidance, social activity guidance, and psychological adjustment. **General post-SPS diet:** High-protein, high-calorie, vitamin-rich, low-fat diet; frequent small meals; reasonable fiber intake; pay attention to food temperature. **For frequent defecation/urgency:** Avoid cold, spicy, or irritating foods. **For constipation:** Increase dietary fiber and ensure fluid intake >2,500 mL/day. **Tool:** Suggested keeping diet and defecation diaries.		**Discharge education (day before discharge, 10–15 min):** Covered postoperative diet, medication, dressing changes, and outpatient follow-up schedule. **Phone interview (within 1 month after discharge):** Routine call to check diet, wound healing, and outpatient visit compliance. Additional patient questions about the disease or treatment were answered accordingly.		**Outcome at 6 months post-SPS:** Bowel symptoms improved significantly in both intervention and control groups. **Effect of self-management programs:** They can promote patients’ self-management behaviors, but their impact on bowel symptoms needs further study.	
**Theoretical basis:** Bandura's self-efficacy theory (four sources: performance accomplishments, vicarious experience, verbal persuasion, and emotional arousal) and the MRC model. **Program structure:** 4-week intensive phase + 8-week maintenance phase (text message-based). **Overall goals:** Improve self-efficacy, bowel function, and HRQOL; reduce healthcare resource utilization in rectal cancer patients undergoing LAR. **Session breakdown:** **Session 1 (before discharge, 30 min, face-to-face):** Education on PFMT, dietary management, and symptom management using tablet with PPT slides. PFMT videos provided to mobile phones for home practice. **Session 2 (first OPD visit after surgery, 30 min, face-to-face):** Introduced a model case, evaluated PFMT/diet/symptom management, and provided feedback. **Sessions 3–4 (3 and 4 weeks after discharge, 20 min, telephone)**: Using semi-structured questionnaires to assess physical/psychological status, provide emotional support, evaluate PFMT performance/difficulty, assess diet/symptom management, and give feedback. **Text message intervention:** Once weekly for 8 weeks to improve PFMT adherence.		**Standard care before discharge:** Educational materials on postoperative care and dietary management. Group education: PPT slides. **Individual education:** Provided by a nurse specialist, covering postoperative diet management and exercise.		**Bowel function:** The program significantly improved bowel function at 3 months post-discharge. **Healthcare utilization:** The experimental group had fewer unplanned pharmacy visits than the control group. **Conclusion:** The bowel function improvement program was effective for male rectal cancer patients in improving bowel function and reducing unplanned healthcare resource use.	

EL, experiential learning; BFI, Memorial Sloan Kettering bowel function instrument; MAPS, motivation and problem-solving; COH-QOL-CRC, City of Hope Quality of Life-Colorectal Cancer; HEI-2015, Healthy Eating Index 2015; PROMIS, Patient-Reported Outcomes Measurement Information System; SQTFFQ, Semi-Quantitative Taiwanese Food Frequency Questionnaire; BSRS-5, Brief Symptom Rating Scale; LARS, low anterior resection syndrome; PANAS-SF, International Positive and Negative Affect Schedule short form; QOL, quality of life; AIMS-RC, altering intake managing bowel symptoms intervention in survivors of rectal cancer; SMART, specific, measurable, attainable, relevant, timely; SPS, sphincter-preserving surgery; BSSBQ, Bowel Symptom Self-management Behaviors Questionnaire; MRC, Medical Research Council; PPT, PowerPoint presentation; PFMT, pelvic floor muscle training; EORTC-QLQ-CR29, European Organization for Research and Treatment in Cancer Quality of Life Questionnaire-C29.

### 3.1 Year and country

The included studies were published between 2021 and 2025. Geographically, the interventions were conducted in the US, China, South Korea, and Denmark (30–34).

### 3.2 Study designs and characteristics

The five included articles were research studies on different nursing-led dietary modification programs for patients with LARS. Four were randomized controlled studies and one was a non-randomized, prospective population-based pilot study. Two were multicenter studies (31, 33).

### 3.3 Patient characteristics

The total sample sizes in the five articles were between 42 and 95 patients. Of the five studies in this review, two included patients with LARS as the target population. One of these studies included patients with minor or major LARS (31) and one only included patients with major LARS (32). The other three articles included patients with rectal cancer following sphincter-saving surgery, regardless of whether they suffered from LARS or not at the time of recruitment (32–34). Li et al.'s (33) sample was limited to patients with low and mid-rectal cancer. Only one study specifically focused on male patients with rectal cancer (34), while the other studies included both men and women. One study enrolled survivors of rectal cancer with a permanent ostomy (31). In the four randomized controlled trial (RCT) studies, the experimental and control groups were homogeneous and no statistical differences in the clinical characteristics between the groups were observed, including the presence of preoperative radiotherapy, diverting stoma, surgical procedure, and anastomosis distance from the anus verge. Thus, there was no selection bias between the groups in these studies. In the single-group pretest-posttest study, the inclusion criteria were clearly defined. However, during the statistical analysis of the intervention effects, no explicit adjustment was made for confounding factors, indicating a possible risk of bias.

### 3.4 Main components, timing, and delivery modes of the nurse-led interventions

The heterogeneity of the dietary modification interventions and the lack of transparency regarding the interventions’ details made it difficult for us to categorize them by intervention content. Of the five articles, two reported interventions that focused only on specific diet-related modifications after sphincter-saving surgery (30, 31). The reported interventions in the other three articles were a combination of several aspects, of which dietary modification was one (32–34).

Despite these differences, we identified and summarized the intervention types based on their main focus. The component details are shown in Table 1.

### 3.5 Intervention development process

The nurse-led interventions reported in two articles were developed using a theory-driven approach (31, 34). Sun et al. (31) developed an intervention program based on the social cognitive theory and the Motivation and Problem-Solving model of behavior change. Kim et al. applied Bandura's self-efficacy theory and the Medical Research Council model to guide the development of their program (34).

Two studies (30, 34) did not specify how their nurse-led interventions were developed. Sun et al.'s (31) intervention program development was not described in detail, but the development process has been published in a previous article (35). Dalsgaard et al. (32) used a multimodal approach that has previously been shown to be effective in the treatment of patients with fecal incontinence using the same personalized nurse-led conservative treatment (36). Li et al.'s self-management program was first developed based on previous studies and a comprehensive literature search, and then a panel of seven experts evaluated the contents of the intervention twice (33).

### 3.6 Form of the dietary modification interventions

The interventions in three of the articles involved initial face-to-face dietary management guidance and telephone call- or email message/phone message-based follow-up (32–34). Tsui et al. (30) conducted one-time nurse-led dietary fiber experiential learning (DFEL) activities at the hospital and face-to-face dietary consultations at the clinic. Sun et al. (31) conducted 10 telephone sessions. Furthermore, three interventions suggested that the patients keep a food diary to help their dietary management and guide discussion on bowel symptom control (31, 33, 34).

The specific dietary modification programs were varied. The dietary management programs comprised the following steps: nurse-led education, dietary management assessment, consultation, further nurse-led suggestions, and reassessment. Three articles described the dietary management methods in detail (30, 31, 33). The nurse-led dietary education in Tsui et al. was conducted using game-based activities, including introducing food categories, dietary fiber, and bowel function, and gaming food selection as assisted by food cards (30). Sun et al.'s intervention focused on bowel symptom-specific dietary management and coached patients on how to accurately document their food intake and note any symptoms associated with the foods. Moreover, SMART (specific, measurable, attainable, relevant, timely) goals for diet behavior change in relation to bowel symptom management were set for their patients (31). Along with the nurse-led sessions, a resource manual was also sent to the patients to guide discussions. Li et al.'s intervention was also symptom-specific; however, patients with different bowel symptoms were categorized into the following two groups: the frequent defecation and urgency group and the constipation group (33). Two studies only mentioned that dietary management advice had been given to patients; however, the specific form of dietary management was not reported. Three studies reported that patients were asked to keep a food diary and monitor which foods aggravated their bowel symptoms in daily life (31, 33, 34).

### 3.7 Content of the dietary modification interventions

Three studies reported the specific content of their dietary modification programs (30, 31, 33). Tsui et al.'s intervention mainly focused on dietary fiber intake management, which included teaching patients about the amount of fiber in different types of foods, how to calculate dietary fiber per gram, and how to choose foods based on daily dietary fiber intake (30). Sun et al.'s intervention mainly focused on coaching patients on how to accurately document their food intake and note any symptoms associated with the foods, and reintroducing foods that were tolerable and beneficial for bowel symptoms. Patients were coached on problem-solving skills to overcome diet behavior change challenges. Throughout the intervention, the skills training was adjusted based on the individual's level of motivation (31). Li et al.'s intervention focused on an individualized diet after sphincter-preserving surgery (SPS) in the postoperative period. This entailed principles such as eating high-protein, high-calorie, vitamin-rich, and low-fat food in the beginning stage after surgery; having frequent small meals; eating a reasonable intake of dietary fiber; and paying attention to food temperature. Patients with frequent defecation and urgency were told to avoid cold, spicy, or irritating foods. Patients with constipation were told to increase their intake of dietary fiber and ensure sufficient fluid intake (>2,500 mL/day) (33).

Four interventions also included psychological and emotional support in the dietary modification process (30, 31, 33, 34). Three interventions were comprehensive nurse-led interventions that included other methods alongside dietary management to control bowel symptoms. These methods were pelvic floor muscle training (32–34), defecation reflex training (33), defecation posture guidance (33), perianal skin management (33), social activity guidance (33), medical guidance (32, 33), toilet routine guidance (32), transanal irrigation (TAI) (32), and biofeedback (32).

### 3.8 Time and duration

The interventions were first delivered before the first discharge after the patient had been diagnosed in two articles (30, 34). Li et al.'s patient self-management program was first conducted 2–3 weeks after surgery (33). Dalsgaard et al. and Sun et al.'s interventions were applied in patients who received treatments after surgery, with a median time from the operation to the first intervention of 13 and 15.9 months, respectively. The duration and frequency of the interventions varied widely, e.g., the intervention duration ranged from 3 to 12 months (32) to 6 months (33), 17 weeks (31), or 12 weeks (34). Tsui et al.'s study did not report a clear intervention duration and only indicated that the intervention had been applied at two timepoints, namely, during the hospital stay and 1 month after surgery (33). Two interventions were delivered in a number of sessions (31, 34). The session lengths in these two articles were 4–93 and 20–30 min, respectively.

### 3.9 Measurement instrument of defecation dysfunction

The low anterior resection syndrome scale (LARSS) was applied in the majority of the studies to evaluate the bowel dysfunction status of the patients after surgery. In Sun et al.'s study, instead of applying the LARSS to assess low anterior resection syndrome, another tool, i.e., the total bowel function score of the Memorial Sloan Kettering Bowel Function Instrument (MSK-BFI), was applied to measure bowel function (31).

### 3.10 Impact of the interventions on defecation dysfunction

Except for in Li et al.'s study, the nurse-led interventions had a significant effect on defecation dysfunction among patients after sphincter-saving surgery. Two dietary modification programs reported an improvement in LARSS score (30, 31). However, the improvement in LARSS score was significant compared to the control group in both the first and third postoperative months in Tsui et al.'s study, while a statistically significant difference in LARSS score was only observed at week 26 between the two groups in Sun et al.'s study. Only one study reported a dose-effect relationship between dietary intake and bowel function, finding that a 1-g increase in fiber intake was associated with a 0.22-point decrease in bowel symptom score (30).

Two studies included dietary management as one part of a comprehensive intervention (32, 34). One of these reported a significant decrease in median LARSS score from 37 to 31 and the prevalence of major LARS fell from 95% to 53% in a group of patients with LARS after the intervention was applied in the clinic (32). Another randomized controlled trial found that their bowel function improvement program was effective in improving bowel function 3 months after discharge. The incidence of LARS decreased from 80.9% (1 month) to 66.7% (3 months) in the experimental group, while the control group showed no change at 95.2% (34).

Li et al.'s individualized self-management program may help prompt patients’ self-management behaviors, but the extent to which they impact patients’ bowel symptoms requires further investigation (33). Finally, the Generalized Estimating Equations (GEE) analysis showed a significant time effect and no group difference in terms of dietary management.

### 3.11 Highlighted suggestions for further development of the interventions

One study did not provide any suggestions for further development or refinement of their intervention. Three studies provided suggestions related to research effectiveness, including extending the follow-up period to further verify the effect (31, 33, 34), increasing the intervention dose (31), and including more research centers (33). They also provided suggestions related to the target population, e.g., expanding the sample size (31, 33), enrolling patients of both sexes (34), and specifically focusing on patients with anastomosis (31).

Other intervention-specific recommendations for refinement were provided, e.g., specific emphasis on healthy eating during coaching sessions (31); developing interventions to improve knowledge application, enhance problem-solving, and strengthen self-learning, and implementing experiential learning activities in post-surgery colorectal care (30); and applying behavioral constructs in health coaching and diet modifications (31). Sun et al.'s study also indicated that the effect of emotional distress on dietary adjustment education and on patients’ bowel symptoms should be investigated (31).

## 4 Discussion

This systematic scoping review aimed to map professional nurse-led dietary management interventions for defecation dysfunction among patients with rectal cancer after sphincter-saving surgery. We included five interventions delivered by nurses in different countries between 2013 and 2025. The studies were published between 2021 and 2025 and included four randomized controlled pilot studies and one population-based, before-and-after pilot study.

The study screening results showed that there were no standard nursing guidelines for dietary modification to help alleviate defecation dysfunction. The number of published nurse-led dietary management intervention studies appears rather limited. Although a significant relationship between dietary intake and improvement in LARS was found in a previous study (23) and dietary self-management has been highlighted as an important method for self-management of LARS by several researchers, studies on the development of dietary modification interventions specifically for LARS management and exploration of the effectiveness of dietary management for LARS are scarce or in their initial stages. There is an urgent need to expand and standardize the dietary modification interventions that are well within the scope of nursing practice.

The evolving nurse-led interventions successfully improved defecation dysfunction symptoms in the four randomized controlled studies included in this review. However, only two of them applied interventions involving dietary modifications alone. It was also found that the nurse-led dietary interventions differed greatly in terms of content, form, and duration. Fiber intake modification was applied in three studies to control bowel symptoms (30, 32, 33). This was also reported in another surgeon-led study (37), which reported the potential positive impact of a high-fiber diet on the gut microbiome and bowel symptoms of patients with colorectal cancer. Fiber has been confirmed by many researchers to be an important factor affecting defecation because it directly affects fecal continence. A prospective cohort study conducted by Staller et al. found that the rate of fecal continence of the participants decreased as the intake of fiber increased (20). High-fiber foods or fiber supplements were found to help control fecal incontinence in another qualitative study (38). Current nursing recommendations for fiber intake modification are varied in terms of consumption standards. High-fiber intake was recommended for patients with constipation in Li et al.’s study, but the other two studies did not mention any specific modification of fiber intake according to specific bowel symptoms. Future research should focus on further developing and testing nurse-led interventions to specifically refine the amount and type of fiber intake to promote the standardization and precision of dietary modification interventions.

Dietary modification needs to be applied according to a dose-effect relationship with bowel symptoms, in line with the current trend of precision nursing (39). However, there are limited studies focusing on dietary intake and specific bowel symptoms among patients after sphincter-saving surgery. The studies focusing on diet self-management are mostly surveys of the subjective experience of dietary influence on bowel symptoms. Trial and error in finding a suitable dietary intake was a common experience among patients. The self-management process lacked scientific rigor and its effectiveness was uncertain. This indicates that the objective relationship between the intake of specific foods and nutrients and specific bowel symptoms and their mechanisms requires further research. Nursing recommendations about dietary intake should be based on the objective impact of dietary intake on defecation dysfunction. The nurse-led dietary modification interventions in this review were developed based on previous studies and literature reviews. Future studies should pay more attention to the original research on the specific relationships between dietary intake factors and defecation dysfunction so that more trials on the effects of precise and scientific nurse-led dietary modifications can be conducted. This is of great significance for constructing a nurse-led dietary management paradigm that can be implemented clinically.

As LARS affects the majority of patients after sphincter-saving low anterior resection to some degree (40) and is more likely to be a chronic condition in patients’ recovery process (16–42), proper nurse-led evidence-based dietary self-management strategies are crucial to relieve bowel symptoms. Nurses have a considerable impact on home care and behavior change throughout patients’ cancer course. In addition, nurses are the healthcare professionals who are in frequent and close contact with patients during admissions. When applying a dietary intervention, nurses need to provide health education in a suitable mode, provide regular remote/face-to-face supervision on dietary modifications, provide feedback about the implementation status of the intervention, and make necessary corrections for the patients (30–34). Therefore, nurse-led dietary management interventions are accessible and essential for patients.

Unlike dietary interventions conducted by dietitians or medical clinics (41) (Mette Borre), nurse-led dietary interventions are preferred when providing holistic care rather than unitary dietary modification. This could be reflected in the studies included in this review. Dietary behavior modification occurs due to a combination of many psychosocial factors. Self-management of diet has been repeatedly confirmed to be an important way for patients to control defecation dysfunction (21–43). Moreover, self-efficacy has been found to be an important factor that influences the effects of dietary modifications. In this review, intervention development was always theoretically guided, using models such as social cognitive theory, the motivation and problem-solving model of behavior change, and Bandura's self-efficacy theory (31, 34). Emotional distress after colorectal surgery was found to be a significant confounding factor for self-management or dietary fiber adjustment education in Tsui et al.'s research, because managing bowel symptoms after colorectal surgery is stressful (30). This was also found in an early study, which found that emotional distress related to incontinence was a barrier to healthcare help-seeking behaviors (44). During the intervention implementation process, emotional support was also an essential aspect of nurse-led dietary management. As our previous qualitative study results found, dietary modification behaviors are influenced by social factors, including the cooking habits of family members, degree of education, and social activities (24). Patients were encouraged to actively participate in social activities and express their feelings to their family and friends to relieve negative emotions (30). Further research studies are needed to further investigate the effect of emotional factors on dietary adjustment education and nurse-led interventions based on the identified relationship between dietary modification behavior and emotional factors could be developed to help control bowel symptoms.

This review reveals that there is a great need to provide interventions that include a longer duration and dose. A more prolonged management intervention may contribute to the patient feeling an increased level of support and reinforce dietary behavior adjustment. The duration and frequency of the interventions varied widely in this review, and only one study started the nurse-led dietary intervention immediately after surgery and continued until 6 months after surgery (33). Two other interventions continued until the first month and third month after surgery, respectively (30, 34). Since defecation dysfunction is the most severe during the first 6 months after surgery and it does not significantly alleviate until the sixth month (45), nursing dietary modification interventions need to cover the first 6 months after sphincter-saving surgery so as to effectively help patients control their bowel symptoms in their time of greatest need. Future studies need to focus on longer nurse-led dietary interventions and to identify a suitable and feasible remote dietary management mode.

This review revealed some deficiencies in the reporting of the interventions. The intervention development process was usually not described in detail. The majority of the studies lacked transparency with regard to the feasibility and acceptability of trials. The specific dietary changes that the patients made were also not sufficiently described. Improving the reporting by sufficiently describing interventions would increase the possibility of adapting or replicating interventions in more research studies (46). Future studies should improve reporting quality in this field to promote the interpretation and translation of this intervention research into clinical practice.

## 5 Limitations

This scoping review identified nurse-led dietary modification interventions for defecation dysfunction in patients with rectal cancer after sphincter-saving surgery. There are several limitations. First, although we applied a broad search strategy and considered nurse-led interventions regardless of the terminology applied, due to the research question, some pertinent articles were omitted from this review, for example, articles that reported dietary interventions conducted by dietitians and doctors. This may preclude the potential for the development of interventions based on multidisciplinary management. Second, due to the limited number of studies in this field, we included studies that conducted dietary interventions only and those that applied comprehensive interventions with a dietary modification component. While this covered a greater number of nurse-led dietary recommendations to map the clear progress in this field, it was not possible to make overall conclusions on the effectiveness of specific dietary modifications. Third, as the aim of this review was to map the literature, the quality of the interventions and articles was not assessed.

## 6 Conclusions

This is the first scoping review to map specific nurse-led dietary modifications and recommendations for the management of defecation dysfunction among patients with rectal cancer after sphincter-saving surgery. In total, five articles were included, of which two articles focused on nurse-led dietary modification interventions only. This review reveals that although the importance of managing bowel symptoms through dietary modification has been identified, research in the field of nurse-led dietary modification interventions to control defecation dysfunction is in the initial stages. This review found that nurse-led dietary modifications showed different degrees of effectiveness in improving defecation dysfunction after sphincter-saving surgery. The interventions in this review were varied in terms of content, mode, duration, and dose and the recommendations were not based on a rigorous level of evidence or standardized. Few studies reported the intervention development process in detail. Psychosocial factors need to be considered in future studies to make dietary interventions more feasible. Bowel symptom-specific dietary modifications also need to be researched in more studies.

Thus, high-quality intervention studies with increased sample sizes are needed that focus on bowel symptom-specific dietary modifications and consider necessary psychological factors to develop evidence-based dietary management interventions. In this way, a more standardized, evidence-based nurse-led dietary modification program could be developed for the management of defecation dysfunction, to promote remote, precise, and scientific clinical nursing care.

## Data Availability

The original contributions presented in the study are included in the article/Supplementary Material, further inquiries can be directed to the corresponding author.
